# Web-Based Nursing Intervention to Promote Physical Activity Among Older Adults After Coronary Revascularization: Protocol for Mixed Method Pilot Study

**DOI:** 10.2196/67678

**Published:** 2025-05-21

**Authors:** Audrey Lavoie, Véronique Dubé

**Affiliations:** 1 Faculty of Nursing Université de Montréal Montreal, QC Canada; 2 Marguerite-d'Youville Research Chair on Humanistic Nursing Interventions Université de Montréal Montreal, QC Canada; 3 Research center Centre Hospitalier de l'Université de Montréal Montreal, QC Canada

**Keywords:** older adults, web-based intervention, coronary heart disease, elder, elderly, older people, physical activities, physical exercise, exercises, exercising, digital, digital health, digital technology, digital interventions, nursing role, nurses, nursing, coronary, cardiology, heart disease

## Abstract

**Background:**

Given the high prevalence of coronary heart disease among older adults and aging populations, there is a need for secondary prevention interventions to help older adults become more physically active. Web-based interventions could be considered for this purpose, knowing that internet use is growing rapidly among older adults. In addition, since older adults would appreciate developing a trusting relationship with a nurse, web-based interventions should include this support, which is not widely observed in the literature.

**Objective:**

This study aims to evaluate a web-based nursing intervention aimed at promoting physical activity in people 65 years and older with coronary heart disease.

**Methods:**

A web-based nursing intervention was developed according to the Intervention Mapping framework in collaboration with a team of health care professionals (n=5) and based on the needs of older adults (n=10). The 7-week intervention (1 session per week) aims to support older adults living with coronary artery disease in resuming, maintaining, or increasing their level of physical activity after coronary bypass surgery or percutaneous coronary intervention. The intervention offers educational content on coronary heart disease and physical activity, suggestions for physical activity, reflective activities, case histories of older adults who have experienced different journeys, an electronic physical activity diary to track progress, and support from a nurse through feedback to increase knowledge, motivation, and sense of self-efficacy. The preliminary effects and impacts of the intervention will be assessed through a mixed method pilot study with a sequential explanatory design. First, a single-group pre-post test will be used to assess the intervention’s preliminary effects on physical activity (electronic journal), quality of life (36-Item Short Form Health Survey version 2), knowledge (quiz), motivation, and self-efficacy (visual analog scale) of 30 older adults living with coronary heart disease, as well as the feasibility of the intervention. Second, a descriptive qualitative design will use semistructured interviews to assess the intervention’s impacts as perceived by 8-12 older adults and its acceptability. Quantitative data on the effects of the intervention will be integrated with the collection and analysis of qualitative data to assess the impact perceived by older adults, using matrices. Nonparametric statistics and a thematic analysis will be produced. A joint display will be used to integrate mixed data.

**Results:**

The results of this study will provide insight into the preliminary evaluation of a web-based nursing intervention to support older adults living with coronary heart disease as they increase their physical activity levels. The recruitment commenced in June 2024, and data collection should be completed by June 2025.

**Conclusions:**

With the potential to promote older adults’ health, this study could guide the development of new interventions to meet the needs of an aging population.

**Trial Registration:**

ClinicalTrials.gov NCT06197347; https://clinicaltrials.gov/study/NCT06197347

**International Registered Report Identifier (IRRID):**

DERR1-10.2196/67678

## Introduction

### Background

Coronary heart disease (CHD) is increasingly prevalent among older adults [1]. While aging contributes to CHD due to arterial stiffness and fatty plaque buildup, secondary prevention strategies, including lifestyle modifications such as increased physical activity, can improve survival and quality of life in this population [2-4]. Increasing physical activity is a core component of secondary prevention, and one of the most important habits to change due to its direct association with a reduced risk of complications and mortality associated with CHD [5-8]. Physical activity has many benefits for the health of older adults, such as improving quality of life [2,9]. Furthermore, such individuals consider an improved quality of life as a priority recovery goal following a coronary event [10,11].

In this regard, secondary prevention interventions have been shown to help promote healthy lifestyles in people with CHD, especially after cardiac revascularization (ie, coronary bypass surgery or percutaneous coronary intervention) [2,9]. However, these interventions, such as cardiac rehabilitation (CR) programs, are underused [12], especially by older adults [13,14], partly due to accessibility issues [15] and their lack of adaptation to aging-related challenges such as comorbidities, frailty, or physical limitations [13]. Given the paucity of literature on the needs of older adults for secondary prevention of CHD after cardiac revascularization, it is essential to explore this subject to develop new accessible interventions that are suitable for this population.

Home-based secondary prevention interventions offer a promising alternative to traditional CR programs, showing comparable benefits in promoting healthy lifestyle habits [16,17]. However, their implementation faces barriers such as high costs [9] and limited human and financial resources in the health care system [18]. The COVID-19 pandemic has further highlighted the need for innovative remote interventions to ensure continued support for older adults [19].

Advances in eHealth technologies (ie, the delivery of health care and services using information and communication technologies, including the web) are resulting in increased use [20]. Web-based interventions can be defined as care or treatments aimed at promoting behavior change and delivered, via a web browser, over the internet through various technological tools such as computers, tablets, and smartphones [20]. Web-based interventions could be considered as a means to support older adults with CHD in their adoption of healthy lifestyle behaviors because of their accessibility and flexibility of use [21], and considering that use of the internet among older adults has increased significantly in recent years [22]. In Canada and the United States, nearly 80% of older adults used the internet in 2022, compared with 73% in 2020 [23,24]. Several web-based interventions are available in the literature, but these are aimed at a population of all ages and none have been developed specifically for older adults [25,26]. Older adults may have distinct secondary prevention needs compared with younger adults (eg, information they want, how they should manage their complex condition, and strategies to maintain their various physical capacities) [27,28] because of the heterogeneous changes that occur with aging (eg, decreased functional capacity, comorbidities, and frailty) [29,30]. Several authors argue that web-based secondary prevention interventions should be developed to address the challenges associated with aging and to respond to the individualized needs of older adults [3,31]. Web-based secondary prevention interventions for people with CHD tend to be tailored rather than individualized to needs [32] and often lack interaction with health care professionals [33,34], factors that are critical for sustained engagement [35,36]. This situation calls for new studies.

Nurses could be well-suited to support older adults through web-based interventions, given the key role they play in managing chronic diseases, including CHD, promoting health and supporting healthy lifestyle habits [37,38]. Their strong interpersonal skills, highly valued by older adults [35,39,40], further reinforce their suitability for this role. Furthermore, their involvement in the secondary prevention of CHD is well-known in the literature [41,42]. To promote behavior change, web-based interventions should also be designed to address key factors influencing the target population, such as motivation and self-efficacy [43,44]. Older adults with CHD can have little motivation to change, low confidence in their ability to become active because of diminished physical abilities related to age [10,45], and may be poorly informed about the benefits of secondary prevention [45,46]. Despite this evidence, no existing web-based intervention has been specifically designed for this population while integrating nursing support. This study aims to address this gap by developing a web-based nursing intervention individualized to the needs of older adults with CHD and that aims to sustain their knowledge, motivation, and self-efficacy toward physical activity.

### Aim and Research Questions

The purpose of this study is to evaluate a web-based nursing intervention to support older adults living with CHD in engaging in physical activity. This study aims to answer the following questions:

- Primary research question
  What is the acceptability (content, structure, and usefulness) and feasibility (recruitment, retention, adherence, and fidelity) of a web-based nursing intervention to support older adults living with CHD in engaging in physical activity?
- Secondary research questions
  What are the preliminary effects of the web-based nursing intervention on the physical activity level and quality of life of older adults living with CHD?
  What are the qualitative impacts of the web-based nursing intervention as perceived by older adults on their physical activity level, quality of life, motivation, knowledge, and self-efficacy?
  How do the qualitative findings on perceived impacts from older adults post intervention complement and illustrate the preliminary quantitative effects of a web-based nursing intervention developed for this population?

## Methods

**Figure 1:**
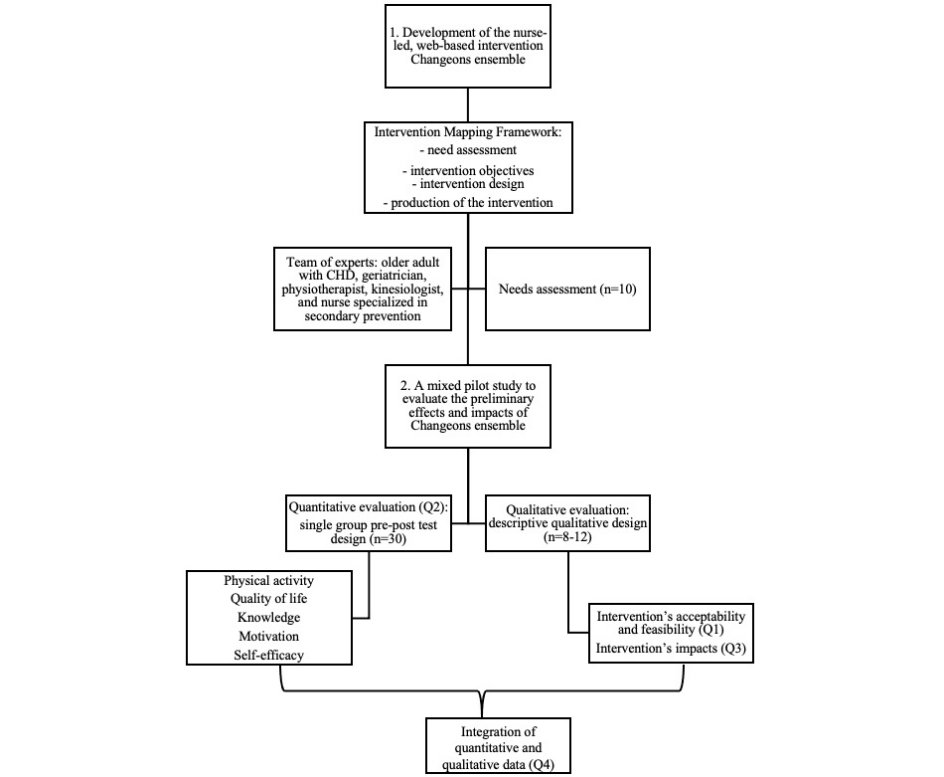
Summary of research protocol. CHD: coronary heart disease.

This protocol is registered on ClinicalTrials.gov (NCT16197347). Figure 1 summarizes the protocol steps.

### Intervention

The nurse-led web-based intervention, named “*Changeons ensemble*,” was designed to support older adults living with CHD who have recently undergone cardiac revascularization in engaging in physical activity. The intervention was developed by the student researcher (AL), her supervisor (VD), and a team of experts (ie, older adult with CHD, geriatrician, physiotherapist, kinesiologist, and nurse specialized in secondary prevention), following the Intervention Mapping approach [44], which includes a needs assessment of older adults living with CHD who have recently undergone cardiac revascularization (n=10). The Intervention Mapping framework is relevant to this study, as it offers a model for intervention development in health promotion that includes all the decision-making steps for intervention planning [44]. Using this framework enabled a systematic approach to intervention development based as much on data collected from a population as on empirical data.

**Figure 2:**
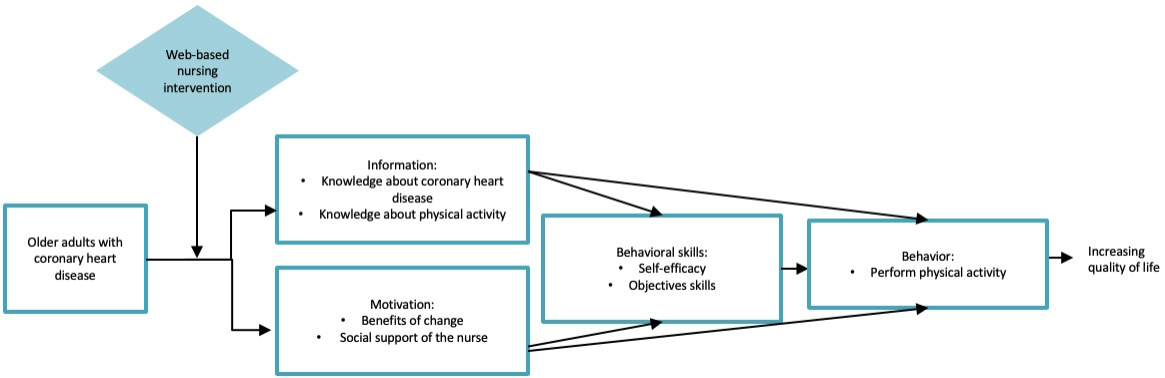
Information-Motivation-Behavioral skills (IMB) model adapted for use in this study.

The intervention is based on the Information-Motivation-Behavioral skills (IMB) model. The IMB model posits that an individual with the necessary information and a high level of motivation can apply behavioral skills (ie, self-efficacy) to achieve behavior change [47]. This model has been selected because it can be used to act on the factors that seem most likely to influence the behavior of older adults, such as knowledge, motivation, and self-efficacy [45], as these factors converge with the constructs of the model. Figure 2, which was adapted with the authors’ permission [48], presents the IMB model and the relationships between the constructs.

Through the intervention, information on physical activity and CHD, such as recommendations, benefits, and impacts, is provided to increase older adults’ knowledge. The intervention aims to bring out the person’s motivation by providing the support of a nurse and by exploring and reinforcing the benefits of change. Vicarious experience, verbal persuasion, and reinforcement of past and current successes are strategies used within the intervention to promote self-efficacy [49].

The team of experts was consulted to provide feedback on the intervention’s content based on their area of expertise. The older adult with CHD offered his opinion on the usefulness and relevance of the intervention’s content and components to be consistent with the needs of the target population. The geriatrician’s contribution was to ensure that the intervention was aligned with the various challenges that can arise with aging, while the physiotherapist offered his expertise on functional limitations and physical activity. The kinesiologist’s role was to validate the examples of suggested exercises for participants, ensuring they were safe in relation to CHD. The nurse ensured that the content was in line with clinical guidelines for older adults living with CHD.

*Changeons ensemble* is a 7-week, nurse-led, web-based intervention that includes educational content (eg, recommendations on physical activity and CHD), reflexive activities (eg, identifying motivation for physical activity), case stories (eg, different pathways of older adults living with CHD who engage in physical activity), physical activity suggestions (eg, instructions and images of cardiovascular or balance exercises), forums (eg, ask questions and exchange with peers), goal planning (eg, a target to be reached each week, with an action plan to achieve it), and an electronic physical activity diary. With a new session available every week, participants log in with a username and password on a secure Moodle (Martin Dougiamas) platform, accessible 24/7 at a time convenient to them. The intervention proposes an initial meeting in person, via videoconference, or by phone with the nurse to foster the older adult’s commitment to the intervention and help build a relationship of trust [40,50]. Every week, the nurse supports participants through the web-based intervention by monitoring their progress, providing asynchronous written feedback by responding to their answers to reflective activities, and facilitating discussions and answering questions on the forum. In this role, the nurse aims to value participants, empower their abilities, bring out their motivations, highlight their progress, and offer advice and encouragement to promote physical activity, quality of life, knowledge, motivation, and self-efficacy. An email reminder is sent to participants after 1 week of inactivity on the platform.

Given the variability in activity levels within this population, the intervention focuses on promoting regular physical activity and reducing postrevascularization barriers rather than prescribing a specific type, duration, or intensity of exercise. We rely on individual support from the nurse and the reinforcement of knowledge, motivation, and a sense of self-efficacy to encourage physical activity, according to each older adult’s physical and health condition.

The intervention was designed with patient-partner and user-friendly principles tailored to older adults, including a simple navigation system, large print and icons, and color contrast. The nurse provides technological support to participants, including explaining how the platform works and assisting them with any difficulties they may encounter. Step-by-step written instructions are provided for additional support.

The Consolidated Standards of Reporting Trials eHealth CONSORT-EHEALTH (Consolidated Standards of Reporting Trials of Electronic and Mobile Health Applications and Online Telehealth) was applied to ensure that all the aspects of the web-based intervention have been reported [20] and is available in Multimedia Appendix 1.

### Design

A pilot study with an explanatory sequential mixed design (quantitative→qualitative) [51,52] will be conducted to evaluate the intervention’s preliminary effects and impacts. The mixed sequential explanatory design implies that a quantitative method is followed by a qualitative one [51,52]. Therefore, the quantitative method for evaluating the preliminary effects of the intervention will be carried out first, followed by the qualitative method for evaluating the benefits perceived by older adults as a result of the intervention.

#### Quantitative Component

For the quantitative component, a single-group pre-post test design will be used to evaluate quantitatively the preliminary effects of the intervention on physical activity level, quality of life, motivation, knowledge, and self-efficacy. This quantitative component is presented according to the SPIRIT (Standard Protocol Items: Recommendations for Interventional Trials) checklist [53], presented in Multimedia Appendix 2.

#### Qualitative Component

For the qualitative component, a descriptive qualitative approach will be used to assess the acceptability and feasibility of the intervention, as well as its impact, as perceived by the older adults on their level of physical activity, quality of life, knowledge, motivation, and self-efficacy. The COREQ (Consolidated Criteria for Reporting Qualitative Research) checklist is presented in Multimedia Appendix 3 and will ensure that the qualitative portion of the study is explicitly reported [54].

### Setting and Sample

Participants will be recruited in the cardiac units of a French-language university hospital in Montreal (Canada). The intervention will be delivered on the open-source learning platform Moodle [55].

#### Quantitative Component

To assess the preliminary effects of the intervention, we will seek to enroll 30 older adults living with CHD. According to some authors, 30 participants are sufficient for a pilot study [56,57], and this sample size is found in similar studies [58,59]. Given that we are interested in a vulnerable clientele that has a low participation rate in CR programs [14] and in research projects in general [60], we anticipate some challenges in terms of both recruitment and retention of participants. As the primary aim of this study is to explore preliminary effects, acceptability, and feasibility, a larger sample size was not deemed necessary at this stage.

Older adults will be recruited by the student researcher by convenience sampling during their hospitalization for coronary bypass graft surgery or a percutaneous coronary intervention. The first author (AL) will screen each patient’s record to verify eligibility and then meet with the eligible patients to evaluate their interest in participating in the project after their hospitalization. Eligibility criteria for participation in this study will be (1) 65 years or older; (2) agreement to participate in the study within 3 months post hospitalization for a coronary bypass graft surgery or 1 month for a percutaneous coronary intervention; (3) no concurrent involvement in an intervention designed to increase their level of physical activity (eg, a CR program, consultation with physical activity expert) during their participation in the project; (4) fluency in French, spoken and written; (5) access to a computer or a tablet connected to the internet; and (6) no cognitive impairments according to the patient record (ie, decreased or impaired complex attention, executive functions, learning abilities, memory, or social skills). Recruitment will take place from June 2024 to June 2025.

#### Qualitative Component

We will contact the 30 older adults who took part in the intervention by phone to invite them to participate in the qualitative evaluation, with the goal of recruiting 8-12 participants. As suggested by several authors [61,62], we estimate that we will need 8-12 participants to reach data saturation regarding their perceptions of the intervention’s impacts and its acceptability. Since the study requires a long-term commitment from the participants, we may not be able to reach the desired number of participants for the qualitative analysis. These results will form part of the assessment of the project’s feasibility.

### Data Collection

The data collection period is planned from June 2024 to June 2025. Table 1 summarizes the study variables and measures, which are discussed in detail in this section.

Electronic physical activity diary inspired by the IPAQ [63].

**Table 1:** Summary of study variables and measurements.

Study variable	Data collection tool	
	Quantitative component	Qualitative component
Sociodemographic data	Self-administered questionnaire	Self-administered questionnaire
Intervention’s acceptability	—^a^	Semistructured interviews with questions on appreciation of the intervention’s content and structure
Intervention’s feasibility	—	Logbook with data (%) on recruitment, retention, adherence, and fidelity to the intervention
Physical activity	Electronic physical activity diary inspired by the IPAQ^b^ [63]	Semistructured interview
Quality of life	SF-36v2^c^ questionnaire: 36 items with 3-, 5- and 6-point Likert scales [64,65]	Semistructured interview
Knowledge	Quiz consisting of 5 multiple-choice questions	Semistructured interview
Motivation	Visual analog 10-point Likert scale	Semistructured interview
Self-efficacy	Visual analog 10-point Likert scale	Semistructured interview

^a^Not applicable.

^b^IPAQ: International Physical Activity Questionnaire.

^c^SF-36v2: 36-Item Short Form Health Survey version 2.

#### Quantitative Component

For the quantitative component, we will assess the preliminary effects of the intervention on the participants’ physical activity level, quality of life, knowledge, motivation, and self-efficacy (question 2). For sociodemographic data, a questionnaire will be completed by the participants at T1 (preintervention).

#### Physical Activity

Participants’ physical activity level will be measured at T1 and T2 (postintervention) via an electronic physical activity diary completed by the participants on the platform. A self-reported physical activity diary has been shown to be easy to administer, well accepted, and more practical than other objective assessments of physical activity, such as accelerometers [66,67]. In this study, the electronic diary was inspired by the French version of the International Physical Activity Questionnaire (IPAQ) [68], by assessing physical activity over the last 7 days (type of physical activity and duration). It enables participants to self-monitor their physical activity each week by entering the type and duration of physical activity performed.

#### Quality of Life

The French Canadian version of a quality-of-life questionnaire (36-Item Short Form Health Survey version 2 [SF-36v2] [65]) will be completed by participants at T1 and T2 via LimeSurvey (Jason Cleeland). This questionnaire has 36 items to answer on 3-, 5-, and 6-point Likert scales, with a higher score indicating a better quality of life. It covers eight concepts: physical functioning, bodily pain, role limitations due to physical health problems, role limitations due to personal or emotional problems, emotional well-being, social functioning, energy, fatigue, and general health perceptions. The SF-36v2 is a revised and improved version of the SF-36 [69]. The SF-36v2 has good psychometric properties with internal consistency reliability ranging from 0.83 to 0.95, test-retest reliability ranging from 0.64 to 0.86, and median correlations of items to their scales ranging from 0.612 and 0.87 [69].

#### Knowledge

Participants’ level of knowledge will be assessed through a quiz to be developed by the research team according to the content of the intervention. The quiz will include 5 multiple-choice questions about physical activity (eg, Which of these answers are benefits of engaging in physical activity?) and will be completed by participants at T1 and T2 via LimeSurvey.

#### Motivation

Participants’ motivation will be assessed at T1 and T2 by auto-captured data on the platform with a visual analog 10-point Likert scale (ie, How important is it for you to increase your physical activity level? 0=somewhat important and 10=extremely important). Visual analog scales are useful for assessing change in individuals over time in a way that is comparable to more traditional Likert-type scales [70,71]. Visual analog scales have been used in several studies to rate subjective feelings about motivation, as they lacked valid rating scales [72-74].

#### Self-Efficacy

As for motivation, participants’ self-efficacy level will also be assessed at T1 and T2 by auto-captured data on the platform using a visual analog 10-point Likert scale (ie, If you decided to increase your level of physical activity, how confident would you be in your ability to succeed? 0=somewhat confident and 10=extremely confident). Visual analog scales have been shown in several studies to be a valid method that allows individuals to visually estimate their sense of self-efficacy [75,76].

#### Feasibility

The following feasibility parameters will be assessed: (1) recruitment (ie, the proportion of eligible patients who agree to participate in the study), (2) retention (ie, the proportion of participants who complete the intervention), (3) adherence (the proportion of sessions and activities completed), and (4) fidelity to the intervention (ie, the difference between the planned and delivered intervention). Due to anticipated recruitment challenges and because the refusal rate during recruitment of older adults generally varies from 20% to 50% [77], we consider it feasible to achieve a recruitment rate of 70% for the desired sample size (n=21 older adults) in the given recruitment time (ie, 1 year). Of this number, we will aim to obtain a retention rate of 75% of the participants for completion of the intervention and data collection (n=16 older adults), considering that a retention rate of about 70%-80% is observed in similar studies of web-based interventions with populations of older adults [36,78,79].

#### Qualitative Component

For the qualitative component, we will assess the acceptability (question 1) and the impacts (question 3) of the intervention. Semistructured interviews will be conducted 1 week post intervention (T2) by a Master’s student, a member of the research team who is unbiased and trained in conducting interviews. The interview guide was developed to address research questions 1 and 3. The acceptability questions were drafted on the basis of the characteristics of an intervention as outlined by Sidani and Braden [80], that is, structure and content. The impact questions were designed to assess the qualitative impact on the various outcomes of the study (ie, physical activity level, quality of life, knowledge, motivation, and self-efficacy). The interviews will last approximately 30 minutes and will be conducted by phone. Acceptability will be assessed through data collected on the intervention’s structure (ie, delivery mode, number of sessions, frequency, and duration) and content (ie, information and activities proposed) to determine whether participants found the intervention appropriate to their situation and relevant in supporting their engagement in physical activity [81]. During the interviews, we will also evaluate the impacts of the intervention, as perceived by the participants, on their physical activity level, quality of life, knowledge, motivation, and self-efficacy (eg, Have you noticed any changes since participating in the *Changeons ensemble* intervention? If so, please tell us about the changes in your physical activity level). The interview guide was pretested with the patient-partner who contributed to the development of the intervention.

### Data Integration (Mixed Component)

**Figure 3:**
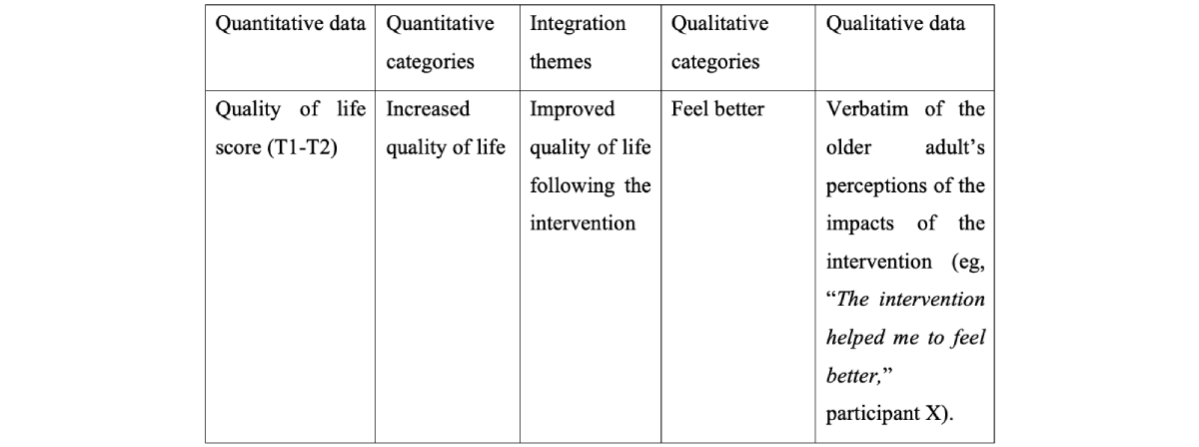
Example of joint display.

For a mixed explanatory sequential design study, some authors propose developing a joint display (refer to Figure 3 for an example). Joint display allows for the presentation of integrated quantitative data (eg, statistical) and qualitative data (eg, verbatim) [82]. In this study, quantitative data on the effects of the intervention will be integrated into the qualitative results [51] of the older adults’ perceptions of the impacts of the intervention. The quantitative results will be integrated into the qualitative results to illustrate how the quantitative effects of the intervention can be connected to the qualitative impacts on physical activity level, quality of life, knowledge, motivation, and feelings of self-efficacy (question 4). To this end, we will follow the four steps proposed by Johnson, Grove, and Clarke [83], which are (1) listing, that is, listing data in the quantitative data (statistics) or the qualitative data (verbatims or categories) column; (2) matching, that is, matching quantitative data with qualitative data based on their similarity, patterns, or parallels; (3) checking, that is, verify whether the data are matched correctly; and (4) pillar building, that is, comparing and contrasting the findings from the last steps to build proposals based on patterns and insights in the integration themes column, to help clarify connections between the data.

### Analysis

The sample will be described using descriptive statistics (eg, mean, SD, percentage, and frequency). Descriptive statistics will also be used to compare, between pre- and postintervention, participants’ level of physical activity, knowledge, motivation, and self-efficacy as well as feasibility data. The Wilcoxon signed rank test will be performed to compare the results from the SF-36v2 questionnaire. Statistical analyses will be performed using QualityMetric’s PRO CoRE system [84]. Semistructured interviews will be recorded and transcribed verbatim, and a thematic analysis [85] will be performed using MAXQDA Analytics Pro (VERBI Software GmbH) 2022. The data will be analyzed by the student researcher (AL) and validated by her supervisor (VD). Themes and subthemes will be identified from the verbatim transcripts and then grouped according to their complementarity, similarity, recurrence, or divergence to form salient thematic clusters. The links between these themes will be further explored to create thematic groupings, and a thematic tree will be developed in the form of a diagram [85].

### Ethical Considerations

This study was approved by the ethics committee of the Centre hospitalier de l’Université de Montréal (23.237) and the Science and Health Research Ethics Committee of Université de Montréal (2024-5351). Written informed consent will be obtained from the participants. The data will be anonymized and stored on a secure server at the research center of the Centre hospitalier de l’Université de Montréal.

## Results

This study will provide a preliminary evaluation of the acceptability, feasibility, impacts, and effects of a web-based nursing intervention for older adults after coronary revascularization. Findings will offer insights into recruitment, retention, and adherence, as well as participants’ perceived impacts regarding physical activity, motivation, self-efficacy, and quality of life. The recruitment commenced in June 2024, and data collection is expected to be completed by June 2025. As of April 2025, we enrolled 20 participants. Results will inform the refinement of the intervention for future studies. Table 2 shows the SPIRIT flow diagram of the study.

**Table 2:** SPIRIT (Standard Protocol Items: Recommendations for Interventional Trials) flow diagram: schedule of enrolment, interventions, and assessment procedures.

Timepoint			Enrolment (–t1)		Preintervention (t1)		Postintervention (t2)
**Enrollment**							
	Recruitment poster	✓					
	Eligibility screen	✓					
	Informed consent	✓					
**Interventions**							
	“Changeons ensemble” intervention			✓		✓	
**Assessments**							
	Physical activity level			✓		✓	
	Quality of life			✓		✓	
	Knowledge			✓		✓	
	Motivation			✓		✓	
	Self-efficacy			✓		✓	

## Discussion

### Implications for Practice and Research

This paper describes the protocol of a study aimed at evaluating a web-based nursing intervention to promote physical activity among older adults with CHD. To our knowledge, this is the first study to propose a nurse-led web-based intervention developed according to the needs of older adults with CHD. This study has important implications for both practice and research.

In terms of practice, the intervention has the potential to promote physical activity and quality of life among older adults with CHD, as well as increase their knowledge, motivation, and sense of self-efficacy. This nurse-led intervention, specifically developed with and for older adults with CHD, could potentially respond to the needs of a sub-group of the population that is often excluded from secondary prevention programs. In terms of the research, this study will generate knowledge about the acceptability and feasibility of this intervention, which could lead to its evaluation on a larger scale and guide further studies in this area. By providing the support of a nurse despite the web-based mode of delivery, this study will generate more knowledge on the role played by nurses in supporting the adoption of healthy lifestyle habits via the web, what has been little studied. However, we believe that this intervention modality may allow nurses to follow older adults than they would in a face-to-face intervention.

### Limitations

This study has certain limitations. First, the design for the quantitative evaluation of the intervention (ie, a single-group pre-post test) implies the absence of a control group. This means that we will not be able to eliminate the influence of confounding variables. For example, our results may be influenced by our participants’ social support networks and digital literacy, which could affect the study’s internal validity. Second, since we are proposing a convenience sampling method and participants may have a high level of digital literacy, the participants we recruit may not be representative of the senior population. As a result, we will not be able to generalize the results, but they will provide new knowledge on the acceptability and feasibility of the intervention, as well as its preliminary effects and impacts on older adults. To encourage the participation of older people with various levels of digital literacy, we made sure to develop an intervention that is easy for older adults to use. Among other things, we incorporated the comments of a patient-partner and took into account factors that may facilitate the use of web-based interventions by older adults (eg, large print and icons, color contrast, simple navigation, etc). In addition, an initial meeting with the nurse (face-to-face, via videoconference, or by phone) will help participants understand how the platform works, making it easier to use. Support will be provided by the nurse throughout the intervention to address any difficulties experienced with technology. Finally, another limitation of this study is the self-reported data, including physical activity levels recorded via an electronic diary. While this approach allows participants to document their activity in a flexible and accessible manner, it is subject to potential recall and social desirability biases. Future studies may consider integrating objective measurement tools (eg, fitness tracker) to complement self-reported measures.

### Conclusion

The global COVID-19 pandemic and the increasing use of the internet by older adults suggest an urgent need to develop new models of care that can be accessed remotely. Such interventions should be capable of being individualized to the varying needs and preferences of older adults with CHD [31]. To facilitate the engagement of older adults in these types of interventions, a relationship of trust with a professional, such as a nurse, would appear to be essential [35,50]. We highly encourage researchers to continue developing and evaluating interventions that promote the health of older adults with CHD.

## Appendix

Multimedia Appendix 1 CONSORT-EHEALTH (v 1.6.1).

Multimedia Appendix 2 SPIRIT (Standard Protocol Items: Recommendations for Interventional Trials) checklist.

Multimedia Appendix 3 COREQ (Consolidated Criteria for Reporting Qualitative Research) checklist.

## Abbreviations

CHD: coronary heart disease
CONSORT-EHEALTH: Consolidated Standards of Reporting Trials eHealth
COREQ: Consolidated Criteria for Reporting Qualitative Research
CR: cardiac rehabilitation
IMB: Information-Motivation-Behavioral
IPAQ: International Physical Activity Questionnaire
SF-36v2: 36-Item Short Form Health Survey version 2
SPIRIT: Standard Protocol Items: Recommendations for Interventional Trials
